# Precision medicine: the foundation of future cancer therapeutics

**DOI:** 10.1038/s41698-017-0016-z

**Published:** 2017-04-24

**Authors:** Seung Ho Shin, Ann M. Bode, Zigang Dong

**Affiliations:** The Hormel Institute University of Minnesota, 801 16th Ave NE, Austin, MN 55912 USA

Based on early evidence of fossilized bone tumors that were found in ancient Egyptian mummies, cancer is an ancient disease.^1^ The term “carcinoma” to refer to cancer was first used around 400 BC by Hippocrates.^2^ The understanding of cancer mechanisms began when John Bennett and Rudolf Virchow observed the abnormal accumulation of white blood cells in patients in 1845, which was one of the first cancers detected by microscopy.^3^ In contrast to the long history of the disease, diagnosis and treatment of cancer at a cellular or molecular level is a relatively new strategy. Although the field of oncology has developed and expanded dramatically, a single drug has not yet been discovered that can cure all patients, even those with similar cancer types. We now know that cancer is an extremely heterogeneous disease, which explains differences not only between cancer cells from different patients, but also between cancer cells within a single patient.^4^ Clearly, more effective strategies are critically needed to defeat the long-standing enemy known as cancer.

The concept and practice of precision medicine is a methodical and systematic movement aimed at defeating diseases such as cancer.^5^ Cancer is a major focus of the precision medicine initiative and developments in precise and effective treatments could benefit many other chronic diseases. Precision oncology or precision medicine of cancer focuses on matching the most accurate and effective treatment to each individual cancer patient based on the genetic profile of the cancer and the individual. Because every single cancer patient exhibits a different genetic profile and the profile can change over time, more patients will benefit if therapeutic options can be tailored to that individual, thus avoiding the idea that “one-size-fits-all” in terms of cancer treatment.

Results of one randomized clinical trial and a small number of feasibility and/or tumor response studies^6–10^ focusing on the concept of precision medicine have had limited success.^11, 12^ Naturally, opponents of precision medicine have criticized the strategy based on the results of the small number of clinical studies.^12, 13^ However, concluding that precision medicine will not work is premature because the precision medicine approach has not yet been fully tested in a sufficient number of trials. In addition, the suggestion has been made that having inadequate access to specific therapeutic agents and an insufficient number of tumor samples may have contributed to the limited success.^13^ Because cancer is a highly heterogeneous disease both between patients and within the same patient, the ability to cope with changes in the clinical trial setting is extremely challenging. Many creative and bold ideas of precision medicine have not yet made the transition from the lab bench to the clinic and need to be more fully evaluated in small clinical studies. For example, the microbiota is now recognized as a key player in health.^14^ The microbiota influences endocrinology and disease status and alters drug response and resistance, and this could hold true for cancer and the efficiency of cancer treatments. Sequencing the human microbiome and modulating the host-microbiota interactions in individual patients may be one approach to improve therapeutic outcomes.^14, 15^

Questions that must be addressed include whether precision oncology is just a theory or whether it realistically assures a better future, and if truly promising how can the application of precision oncology be improved and effectively implemented into the clinic? Several lines of evidence strongly support the idea that precision oncology could likely benefit more patients compared with traditional chemotherapies. First, some patients’ lives have already been substantially improved by target-based therapies compared with conventional cytotoxic therapies. One of the most notable examples is the discovery of the *Bcr-Abl* gene fusion in chronic myeloid leukemia (CML). Uncovering this genetic driver of CML lead to the development of a selective inhibitor of BCR-ABL, imatinib, which exhibited broader treatment coverage because, unlike other gene mutations, the *Bcr-Abl* gene fusion occurs in almost all CML patients. This compound improved the overall survival rates of CML patients to 90% over 5 years and 88% over 8 years.^16^ Another example includes the effectiveness of drugs like trastuzumab, lapatinib, pertuzumab, or ado-trastuzumab emtansine against human epidermal growth factor receptor 2 (HER2)-positive breast cancer. Compared with chemotherapy alone, the addition of trastuzumab to chemotherapy significantly slowed the disease progression (i.e., median, 4.6 vs. 7.4 months), increased the objective response rate (i.e., 32 vs. 50%), prolonged survival time (i.e., median, 20.3 vs. 25.1 months), and reduced the risk of death by 20%.^17^ Lapatinib plus chemotherapy (i.e., capecitabine) achieved a longer median time to disease progression compared with chemotherapy alone (i.e., 8.4 vs. 4.4 months).^18^ A combination of pertuzumab, trastuzumab, and chemotherapy (i.e., docetaxel) further improved the median overall survival time to 56.5 months compared with a combination of only trastuzumab and chemotherapy (i.e., 40.8 months).^19^ Treatment with ado-trastuzumab emtansine, a conjugate of a HER2 monoclonal antibody and a cytotoxic drug, significantly improved the length of progression-free survival and overall survival with lower adverse effects when compared with lapatinib and chemotherapy (i.e., capecitabine).^20^ These two examples demonstrate how the identification of key mutations like the *Bcr-Abl* fusion or HER2 can clearly benefit a larger number of selected cancer patients.

Second, many strategies are now available to identify important molecular targets for therapeutic intervention. Synthetic lethality is one unique strategy directed toward identifying cancer vulnerabilities.^21^ This strategy is based on the discovery that cell death is caused by a combination of deficiencies in the expression of two or more genes, whereas deficiency in only one of these genes can increase viability.^21, 22^ In 2005, the *BRCA* and *poly ADP ribose polymerase* (PARP) genes were found to have a synthetic lethal relationship.^23, 24^ In 2014, the FDA (Food and Drug Administration) authorized accelerated approval of olaparib, a PARP inhibitor, to treat *BRCA*-mutant ovarian cancer patients.^25–28^ Progression-free survival of *BRCA*-mutant ovarian cancer patients was significantly prolonged by olaparib compared with the placebo treatment (i.e., median, 11.2 vs. 4.3 months).^28^ Likewise, several candidate proteins were identified to treat cancers that over-express Myc, a transcription factor for which small-molecule inhibitors are currently unavailable.^29–33^ Within just a 5-year timespan, researchers found that Myc-amplified tumors were sensitive to CDK1, aurora kinase B, and BRD4 inhibition.^29, 30^ Although many of these inhibitors have yet to translate into clinical successes, Phase I trials of three BRD4 inhibitors are ongoing (CPI-0610 and TEN-010) or have just finished (OTX015).^34, 35^ These findings suggest that changes in *gene A* in cancer does not necessarily mean that *gene A* is the best target, but instead targeting its synthetic lethal partner might be a more effective strategy.

Third, clinical trial design is constantly evolving to overtake tumor heterogeneity from patient to patient. The Molecular Analysis for Therapy Choice (NCI-MATCH) is a clinical trial selecting treatments based on genetic features of patients, not traditional tumor histology.^36^ The cancer patients will be assigned to 1 of 25 different treatment arms based on their genetic mutation profile. The overall response rate will be the endpoint to measure success. However, no control arms will be included, which could dramatically affect the interpretation of the final results. Even though a number of questions have been raised, the investigators are very optimistic that the results will further the efforts to implement precision oncology treatments. The Molecular Profiling-based Assignment of Cancer Therapy (NCI-MPACT) is another innovative clinical trial to test the hypothesis that targeting an oncogenic driver mutation is more efficacious than not targeting it. NCI-MPACT will recruit advanced cancer patients who have been unresponsive to standard therapeutic options and possess mutations in one of three genetic pathways that include DNA repair, PI3-K/mTOR (phosphoinositide-3 kinase/mammalian target of rapamycin), and Ras/Raf/MEK (mitogen-activated protein kinase). Patients without a driver mutation will not be eligible for further treatment.^37^ Although this trial is similar to the NCI-MATCH in that patients will undergo tumor biopsies when enrolled, patients in the NCI-MPACT study will be assigned to one of two arms: treatment with drug(s) designed to target the mutation or treatment with drug(s) not prospectively identified to target the mutation. By evaluating gene targets across the histologic subtypes with NCI-MATCH and addressing the importance of driver mutations with NCI-MPACT trials, the efficacy of diagnosis and therapies could be significantly enhanced.

Finally, the influence of new technologies such as the CRISPR/Cas system and cryo-electron microscopy (cryo-EM) will broaden and sharpen our ability to identify novel therapeutic targets for precision oncology. CRISPR/Cas technology enables controlled exchange, insertion and deletion of DNA sequences unlike spontaneous mutation, and can easily generate animal models that mimic mutations status of patients.^38^ Recently, a gene therapy trial to treat myeloma, melanoma, and sarcoma with CRISPR/Cas has been approved by the National Institute of Health and is awaiting approval from the FDA.^39^ In addition to CRISPR/Cas, cryo-EM is a promising tool for precision oncology. Cryo-EM is a type of transmission EM in which samples are examined at cryogenic temperatures.^40^ Because the samples (e.g., proteins and viruses) are frozen in their native states, researchers can study biological events accurately at the subatomic or atomic level. For an instance, a 2.3 Å resolution cryo-EM structure of p97 showed a large corkscrew-like hexameric form, revealed its interactions with an allosteric inhibitor, and displayed conformational changes induced by adenosine tri-phosphate.^41^ Visualization of intact proteins and anti-cancer drugs at subatomic or atomic levels will assist researchers in understanding the consequences of genetic alterations on drug response and resistance.

In spite of some early setbacks, precision oncology still has a great deal of promise and should not be abandoned hastily. The challenge of tumor heterogeneity should not discourage or intimidate efforts to overcome cancer but should push the field forward. As practice makes perfect, precision mends patients.
